# Supplementary material for: Detection of malaria infection from parasite-free blood smears
Source: Malar J. 2026 Jul 17;25:362. doi: 10.1186/s12936-026-06047-z (PMC13660055; doi:10.1186/s12936-026-06047-z)
Supplement: Supplementary file 1 [file 12936_2026_6047_MOESM1_ESM.docx]

# Supplementary Information

## Supplementary Figures

**Supplementary Figure 1:** **Representative raw thick and thin blood smear slides, together with high-magnification fields of view used for model training and evaluation** **a.** 3 examples of thick (left side of the slides) and thin (right side of the slides) blood smear slides stained with Giemsa **b.** Random fields-of-view (FoVs) acquired from thin blood smears at ×1000 oil immersion magnification using a smartphone-mounted microscope for each diagnostic class: *submicroscopic* (bottom row), and *negative* (top row). S*ubmicroscopic* samples contain no visible parasites despite qPCR positivity, and *negative* samples are both microscopy and qPCR-negative. These images highlight the challenge of identifying submicroscopic infections through human inspection alone.

**Supplementary Figure 2:** **Confusion matrices for the best model** — selected based on accuracy and sensitivity — across 4-fold cross-validation. Matrices are shown per fold, each evaluated with a distinct random seed.

**Supplementary Figure 3:** **Receiver Operating Curves for the best model** — selected based on accuracy and sensitivity — across 4-fold cross-validation. Curves are shown per fold, each evaluated with a distinct random seed.

**Supplementary Figure 4:** **Accuracy on FoV number curves for the best model** — selected based on accuracy and sensitivity — across 4-fold cross-validation and 4 different seeds. Curves are shown per seed, each evaluated on the 4 folds.

**Supplementary Figure 5:** **Empirical Cumulative Distribution Functions for the top 10 features with highest KS statistic.** ECDF plots with related KS stat & p-value are computed per fold on true data samples.

**Supplementary Figure 6:** **Statistical ranking of morphological features distinguishing true samples.** Features are ranked by their Kolmogorov–Smirnov (KS) statistic (blue bars, left axis), representing the magnitude of the difference between negative and submicroscopic sample distributions. The corresponding p-values (red line, right axis; logarithmic scale) indicate statistical significance. The horizontal dashed green line denotes the Bonferroni-corrected significance threshold ($P < 9.43 \times 10^{-7}$), adjusted for multiple hypothesis testing across $n=106$ features. Features with p-values above this threshold (above the green line) are automatically rejected.

**Supplementary Figure 7:** **qPCR parasitemia against CellProfiler morphological features.** For all slides, segmented single cell CellProfiler features, previously identified as strong morphological markers distinguishing qPCR-negative from submicroscopic-positive slides, were aggregated to slide-level feature values and plotted against parasite density as assessed by qPCR ; all qPCR-negative slides are shown at 0 parasites/µl and submicroscopic cases are displayed on a logarithmic parasitemia scale. Each point represents one slide, colored by class. Dashed orange lines display the fitted linear trend among submicroscopic slides, and we report Spearman correlations both for all slides and submicroscopic subset.

## Supplementary Tables

**Supplementary Table 1:****. Performance metrics for models using *simple* and complex (last four rows) slide-level aggregation.** Reported accuracy corresponds to the overall mean across 4 stratified folds of the per-fold mean accuracy over 4 runs with distinct random seeds. Values are presented as mean ± std.
